# The Effects of Sports Vision Training on Binocular Vision Function in Female University Athletes

**DOI:** 10.1515/hukin-2015-0131

**Published:** 2015-12-30

**Authors:** Teresa Zwierko, Lidia Puchalska-Niedbał, Justyna Krzepota, Mikołaj Markiewicz, Jarosław Woźniak, Wojciech Lubiński

**Affiliations:** 1 Department of Physical Culture and Health Promotion, University of Szczecin, Szczecin, Poland; 2 Department and Clinic of Ophthalmology, Pomeranian Medical University, Szczecin, Poland; 3 Institute of Vision Training and Therapy, Warszawa, Poland; 4 Institute of Mathematics, University of Szczecin, Szczecin, Poland

**Keywords:** orthoptic exercise, oculomotor function, sport training

## Abstract

Binocular vision is the most important visual cue for spatial orientation in many sports. In this study, we investigated how binocular vision was influenced by an eye training program that may be used to improve individual's oculomotor function. The experiment involved twenty-four female student athletes from team ball sports (soccer, basketball, handball). After an initial testing session, 12 participants were randomly allocated to the experimental group. Optometric investigation which included synoptophore testing and a test of dissociated horizontal phoria based on the Maddox method was performed three times: before the experiment, after eight weeks of eye training (3 times a week for 20 minutes), and four weeks after the experiment was terminated. Eye exercise methodology was based on orthoptic, sport and psychological aspects of performance. The phoria screening examination showed that exophoria was the most frequent disorder of binocular vision. Low fusional vergence range was also observed. Following the training period, 3 of the 6 oculomotor variables improved. The greatest effect was observed in near dissociated phoria (χ^2^ =14.56, p=0.001 for the right eye; χ^2^ =14.757, p=0.001 for the left eye) and fusional convergence (χ^2^ =8.522, p=0.014). The results of the retention test conducted four weeks after the experiment confirmed the effectiveness of the vision training program. The results of the study suggest that binocular functions are trainable and can be improved by means of appropriate visual training.

## Introduction

In many sports binocular vision is the most important visual cue for spatial orientation, as it enables athletes to extract precise information about the locations of objects in three-dimensional (3D) environments (Jackson et al., 1997). It is crucial for athlete's perception in both static and dynamic situations in regard to the position of the target (e.g. ball), as well as the speed and the distance of the target (Coffey and Reichow, 1990; Bauer et al., 2001; Laby et al., 2011). Normal binocular vision requires accurate alignment of the eyes and binocular mechanisms for vergence function, sensory fusion and stereopsis (Thorn et al., 1994).

It has been reported that highly developed binocular vision function enhances performance of motor skills. For example, Lenoir et al. (1999) compared one-handed catching performance between catchers with high and low binocular depth vision or stereopsis. In their study, tennis balls were projected at three different velocities towards the subject. Participants with better stereopsis were more successful at catching the balls compared to those with poor binocular vision function. The participants with low stereopsis made more temporal errors. As the velocity of the ball increased, initiation of the catch was delayed and catching performance decreased. This observation was confirmed by Mazyn et al. (2007) who showed that catchers with a lack of stereopsis had only a moderate or a non-significant improvement in catching performance after intensive training (more than 1400 trials), while the group with good stereo vision improved from a catching percentage of 18 to 59%.

Several studies of athletes’ vision demonstrated superior stereopsis performance in sports which require rapid and accuracy visuomotor function. An early study by Solomon et al. (1988) indicated significant differences in dynamic stereopsis between baseball players and untrained subjects. Other study results confirmed that professional baseball players had significantly better distance and near stereo acuity (as a quantitative measure representing the minimum disparity that can still be correctly identified) than those of the general population (Laby et al., 1996; Boden et al., 2009). Furthermore, Coffey et al. (1994) analyzed stereopsis response times when comparing professional golfers with amateur and senior ones. They observed superior response times for professional athletes. Laby et al. (2011) described the visual functions of Olympic-level athletes and confirmed some differences between particular sports disciplines. They observed, among others, that the mean distance contour stereo acuity of archers was worse than of soccer, softball and speed-skating athletes. The results indicated that a unique set of visual skills is common to athletes in certain sports. However, a recent study by Paulus et al. (2014) demonstrated that professional and amateur soccer players did not show superior results in computer-supported stereopsis tests, including stereo acuity and response times, compared to untrained subjects. The soccer players showed significantly superior choice reaction times only for monocular stimuli. These apparently contradictory results may be associated with non-standard techniques of stereopsis evaluation applied by those authors.

Significant interest exists regarding improving sports performance by using training procedures to enhance vision skills. Previous research (Quevedo et al., 1999; Abernethy and Wood, 2001; Zupan et al., 2006; Maman et al., 2011; Rezaee et al., 2012) has shown mixed training effects on different aspects of visual ability in sport, which can mostly be attributed to the use of different training program methodologies (Schwab and Memmert, 2012). This situation may be associated with the existence of relatively few theoretical concepts describing the methodology of visual training (Helveston, 2005). Erickson (2007) concluded that the most important requirement of selecting training procedures is to identify those visual abilities which are critical to athletic performance and use vision evaluation and training systems directly related to specific task demands in the particular sports disciplines. Therefore, we proposed a program for team games that addressed athletes’ eye alignment as well as motor fusion evaluation and training on stimuli for far and near vision. The training procedure aimed to improve the ability to rapidly adjust focus and eye alignment for variety of fixation distance taking into consideration individual athletes’ binocular system disorders.

Binocular single vision is achieved by the coordinated synergistic movement of both eyes. The relative deviation of the visual axes is known as heterophoria. There are a variety of heterophoric deviations. If the visual axes converge, the condition is called esophoria, and if they diverge, it is known as exophoria. Most investigations have found some degree of heterophoria in 70–80% of the population (Kommerell and Kromeier, 2002; Perz et al., 2010). Proper alignment of the eyes is guaranteed by a normally functioning fusion mechanism. Fusion consists of motor (vergence) and sensory components. Motor fusion is induced via the motor system of the eyes. In the absence of a properly functioning fusion mechanism, a more or less obvious deviation of the visual axes will be present. Several experimental studies have shown that the extraocular muscle tone changes produced by altering the amount of heterophoria result in a distortion in perceived distance, i.e. an esophoric shift causes an increase of perceived distance, and an exophoric shift induces shortening of perceived distance (Coffey and Reichow, 1990). It seems that correct alignment of the two eyes is critical in team games requiring accuracy when aiming at a stationary target (i.e. a free throw in basketball), as well as in situations involving fast-moving objects (i.e. passing and receiving a ball). It is also indicated that misalignment of the eyes can make an athlete adopt an improper stance, posture or body position causing a deterioration in technique in order to compensate for the visual problem (Ericson, 2007).

The present study aimed at investigating the efficiency of an eye training program used to improve an individual's ability to align their vision in relation to the demands of the sport. We tried to create a methodology of exercise based on orthoptic, sport and psychological aspects of performance. The main objectives were to (1) assess binocular vision based on an objective ophthalmological and optometric measurement of selected variables characterizing the function of the visual system, and (2) determine whether (and if so, to what extent) orthoptic exercise could improve ocular alignment and motor fusion. We hypothesized that an eye training program would improve binocular function in athletes with identified visual deficiencies in ocular alignment and motor functioning of the eyes.

## Material and Methods

### Participants

Twenty-four female athletes from the Szczecin University (21.55 ± 0.67 years) involved in team sports (soccer n=8, basketball n=8, handball n=8) participated voluntarily in the experiment. Mean sport experience was 9.47 ± 4.12 years. Multiphase sampling was used to gather the data. Part of the information was collected from the whole sample and part from a subsample. After an initial testing session, 12 athletes were randomly allocated to the experimental group. All participants of the study were healthy, none had a history of past ocular treatment or had taken part in prior vision research. The ophthalmological tests included: distance visual acuity, refractive errors, the anterior segment and fundus of the eye examination, accommodation, the eye position, the study of the eye motility into each of the six cardinal positions of gaze. There were three grades of binocular vision with synoptophore testing: simultaneous perception, fusion and stereopsis (stereoscopic vision with dissociated pictures was examined). The optometric measurement revealed that all the subjects had binocular vision in free space and on synoptophore: simultaneous perception in 100%, sensory fusion in 100% and stereopsis in 95.8% (lack of the stereopsis was foumd in one person). The ophthalmologic and optometric investigations were carried out in the Department and Clinic of Ophthalmology at the Pomeranian Medical University. Subjects gave their written informed consent and were allowed to withdraw from the experiment at any time. The procedures followed in the study were approved by the Ethics Committee of the Regional Medical Chamber in Szczecin (Approval number11/KB/V/2013).

The optometric study included:

1. Eye’s alignment: a screening test of dissociated horizontal phoria based on the Maddox method (for far 5 m – a Maddox rod, for near 30 cm – a Maddox wing). In this procedure the room light was dimmed. For far phoria testing, a single Maddox rod (series of red cylinders) was placed horizontally in front of the right (left) eye, and the subject fixated on a distant spot of white light. The participant saw a vertical red line and a white spot. The subjects were asked to report the relative position of the Maddox rod streak with respect to the white light. If there was no phoria, the line passed directly through the spot. If the image was crossed (i.e. the line was to the left (right) of the light), exophoria was indicated; if the line was to the right (left), it indicated esophoria. The angle of eye deviation was identified on the Maddox scale. For near, a Maddox wing was used. For near phoria testing, a respectively smaller scale was applied. The procedure was the same as in distance phoria testing.
2. Motor fusion: a synoptophore (Sbisa Industriale SpA) was used for diagnostic fusion range. The synoptophore consisted of a base which connected horizontally movable arms. The base also contained chin and forehead rests and buttons for adjusting the intensity and frequency of light sources placed in the movable arms. The rotation angle of the arm was read from a scale in degrees of arc (1 deg of arc is approximately 2 prism diopters (PD). The subject was supposed to properly connect similar pictures. A positive range of fusion (convergence) with moving arms nasally and negative range of fusion (divergence) with moving arms temporally were measured. We looked for so-called break points. The range of fusion should be greatest in the convergence direction and smallest in the divergence direction. For adults, the normal fusional amplitude is 25 PD for convergence and 10 PD for divergence.

### Procedure

The experimental group participated in a special training program to improve their visual ability over eight weeks, 3 times a week for 20 min. Optometric examination was performed three times: a pre-test (before experiment), a posttest (after eight weeks of experiment) and a retention test conducted four weeks after the completion of the experiment. The training program was controlled by a psychologist and a vision improvement teacher. Orthoptic exercises were administered specifically as:

1. An extraocular muscle warm-up: smooth pursuit eye movements in different directions with closed and opened eyes (central focus, lateral right/left, up/down; upper right/left, lower right/left, circular movements right/left), resting.
2. Horizontal and vertical saccadic eye movements (e.g. searching out numbers in a whole square divided into four quadrants with randomly positioned digits: in order, i.e. 1, 2, 3 etc., or 2, 4, 6, 8 etc., or in other configurations specified by the coach in each quadrant or in the whole square, searching a light signals placed in different distance on the court, incorporating specific movement to the task (e.g. keeping a ball).
3. Smooth pursuit eye movement (e.g. following the partner's finger plotting different patterns in the air – the number eight, the sign of infinity, a circle, a spiral, etc., following the moving object held by the partner using a dice while reading the number of spots on the dice, or a ball with a letter or number written on it).
4. Near-far-near charts were used to improve oculomotor functions. Participants read letters up close (20 cm) followed immediately by letters at 6 m. The participants focused back and forth on the card and counted how many iterations they could do. The cards had rows of random letters so that the subjects had to track their progress in a similar fashion to the saccades.
5. A Brock string, a 3 m (and 6 m) string with 3 colored beads (diameter of 18 mm). The subjects had the nearest end of the string by their nose and had it extended away from them parallelly to the ground. The subject needed to focus on the subsequent beads, set along the string, back and forth for 1 min. We used this exercise with the string under different conditions, e.g. standing, walking, an unbalanced body position, double bead string fixation exercise, etc. This required adaptation and convergence of the eyes to find and focus on the beads. Athletes developed the ability to perceive images from both eyes and to shift fixation quickly and accurately from one point to another.
6. For fusional convergence and divergence training we used exercises with free space fusion cards, eccentric circles cards, variable vectograms, dynamic vision exercises with objects/symbols at different distances: near and far. The subject followed this training with increasing difficulty, e.g. standing with a changing position of the objects, walking, an unbalanced body position, incorporating specific movement to the task (e.g. keeping a ball).
7. Relaxing exercises (relaxation massages, palming).

### Data analyses

Descriptive statistics were performed on all data. The assumption of normality was examined using the Shapiro-Wilk test. The distributions of the samples were not normal, and the Levene test showed that homoscedasticity was also violated. Thus, a nonparametric Friedman chi-square ANOVA test was used for results obtained in the pre-test, post-test and retention tests. Differences were considered as statistically significant at p<0.05. Post-hoc (Wilcoxon signed rank test) analysis was performed with a Bonferroni correction (lowering the threshold of statistical significance to p<0.017). Statistical analysis of data was performed using STATISTICA software, version 10, from StatSoft, Inc. (2011).

## Results

### Effects on eye alignment

The results of initial testing of distance and near dissociated horizontal phoria are presented in Table 1. The most frequent eye position revealed during screening distance horizontal phoria examination was a shift towards exoforia (54.17% for the right eye, 45.84% for the left eye). Ortophoria occurred in 16.67% of cases for the right eye and 29.16 % of cases for the left eye. Screening for near horizontal phoria examination revealed a shift towards exophoria in 83.33 % of cases for the right eye and in 79.17% of cases for the left eye. An examination of distance heterophoria revealed abnormal results in 16.67% of the study participants.

**Figure 1:**
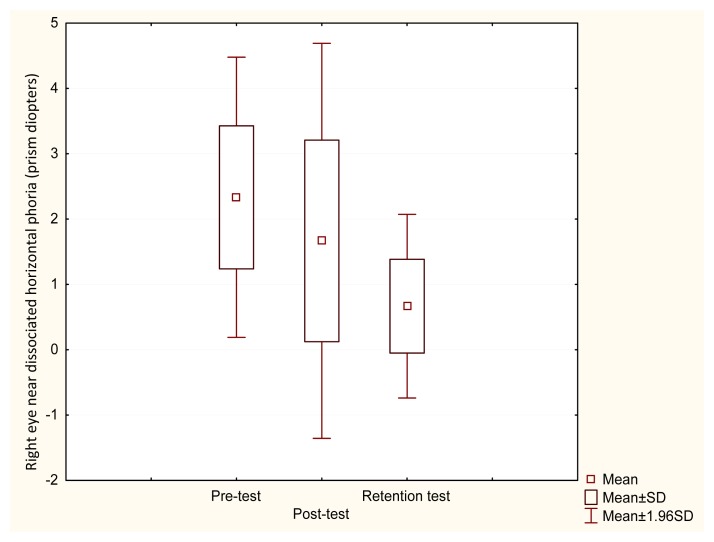
Pre, post and retention test values of right eye near dissociated horizontal phoria in the experimental group. There was a significant difference between pre–test and retention test results (Z=3.059, p=0.002)

**Figure 2:**
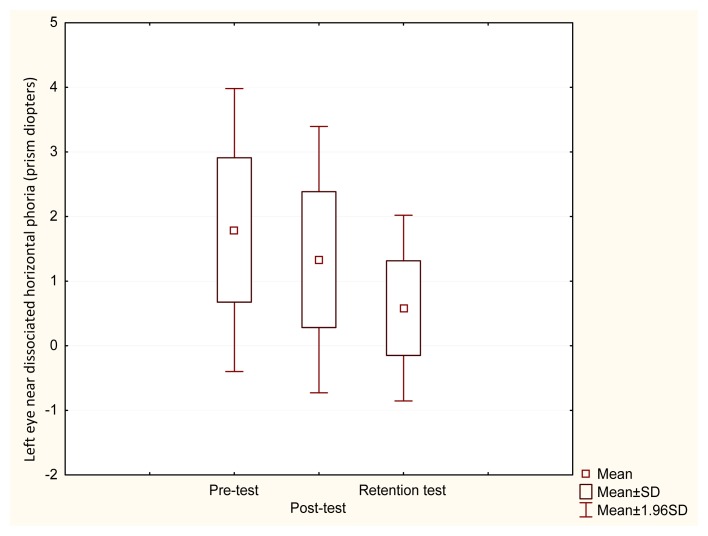
Pre, post and retention test values of left eye near dissociated horizontal phoria in the experimental group. There was a significant difference between pre–test and retention test results (Z=2.934, p=0.003)

In the experimental group abnormal results of distance and near dissociated horizontal phoria in 25% of cases were observed. The results of right eye near dissociated horizontal phoria showed a significant improvement after visual training intervention (χ^2^ =14.56, p=0.001). Post-hoc Wilcoxon test analysis provided no significant difference between pre and post–test results (Z=1.680, p=0.092), however, showed a significant difference between pre–test and retention test results (Z=3.059, p=0.002) (Figure 1). Similarly, left eye near dissociated horizontal phoria analysis showed significantly better results (χ^2^ =14.757, p=0.001). Post-hoc Wilcoxon test analysis presented no significant difference between pre and post–test results (Z=1.606, p=0.108), however, showed a significant difference between pre–test and retention test results (Z=2.934, p=0.003) (Figure 2).

Right eye distance dissociated horizontal phoria results were found to be significantly better after training (χ^2^=8.643, p=0.013). However, the results of post-hoc Wilcoxon test analysis presented no significant difference between pre and post–test (Z=1.944, p=0.052). Pre–test and retention test results showed smaller p (Z=2.201, p=0.028), however, could not be considered statistically significant after using the Bonferroni correction. The left eye distance dissociated horizontal phoria results showed no significant differences between subsequent tests (χ^2^=3.355, p=0.187).

### Effects on motor fusion

The results of the initial testing session of motor fusion are presented in Table 3. The mean results of convergence motor fusion were 12.38 ± 8.20 degrees of arc. In relation to the norm, 58.34% presented results below the normal value. The mean results of divergence motor fusion were 4.74 ± 1.93 degrees of arc. From the entire study population, 41.67% of participants had divergence motor fusion results below the norm.

**Figure 3:**
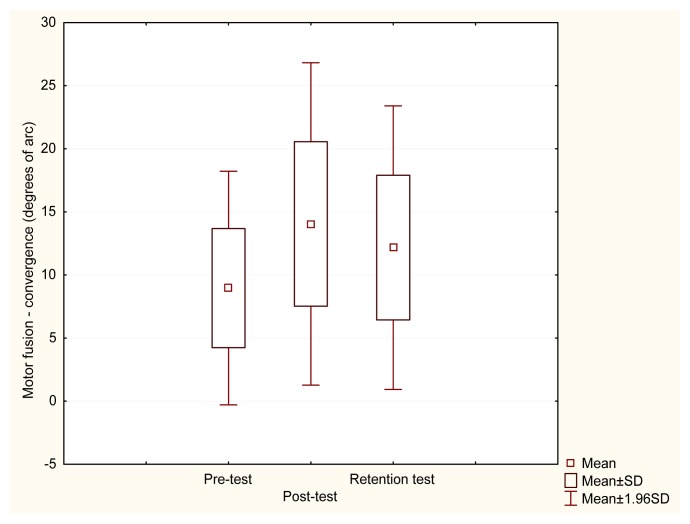
Pre, post and retention test values of the convergence motor fusion in the experimental group. There was a significant difference between pre and post-test (Z=2.628, p=0.009) and pre–test and retention test results (Z=2.294, p=0.022)

After visual training, there was a statistically significant difference observed in fusional convergence results in the experimental group (χ^2^=8.522, p=0.014). The post-hoc Wilcoxon test revealed significant differences between both pre and post-test results (Z=2.628, p=0.009) and pre-test and retention test results (Z=2.294, p=0.022) (Figure 3). To the contrary, no significant difference was found in fusional divergence results (χ^2^=2.513, p=0.285).

## Discussion

In the present study, the effects of participation in 8 weeks of the eye training program on the objective measures of ocular alignment and motor fusion range were investigated. Following the training period, 3 of the 6 oculomotor variables improved; a particularly positive effect was observed for near dissociated phoria and convergence motor fusion. In those cases, the results of the retention test corroborated the effectiveness of the vision training program. In contrary, the training effects for distance dissociated phoria and divergence motor fusion were smaller and not statistically significant.

Our study confirmed that small heterophoria was common in a healthy population. A small deviation expressed in prism diopters (PD) is considered as a physiological range (1 PD to 2 PD of esophoria or 1 PD to 4 PD of exophoria in distance). However, it is indicated that hyperphoria of 1 PD in either eye nearly always produces symptoms, and only 0.5 PD of hyperphoria can be considered to be within the physiologic range (Von Noorden, 1996). About 70% of the study participants had a deviation of the visual axes for distance, and about 80% had a deviation of the visual axes for near. These results are in line with previous research demonstrating that heterophoria is present in 70–80% of the population, in most of cases within the physiological range (Kommerell and Kromeier, 2002). However, Perz et al. (2010) indicated abnormal results in distance horizontal dissociated phoria in 51.4% of cases (74 of 144 healthy students). An examination of distance heterophoria in our experimental group revealed abnormal results in 25% of the participants, with more exophoria. The positive effects of eye exercise training were confirmed in cases of abnormal eye alignment as well as small deviations (smaller effect at distance fixation).

One important factor causing misalignment of the visual axes is a weak vergence system (Wilmer and Backus, 2009; Lavrich, 2010). It should be noted that the clinical significance of heterophoria depends not so much on their absolute values as on the correlated fusional amplitudes (Radaković et al., 2012). Phorias are normally controlled by the fusion mechanism, which is the binocular system's drive to fixate the same object with both eyes via vergence eye movements (motor fusion) resulting in sensory fusion. In the present study, a low value of fusional convergence in the majority of cases was observed.

Fusional convergence amplitudes are the amount of convergence available to overcome temporal disparity in order to maintain binocular fusion. It is generally accepted that normal fusional amplitudes for adults consist of 25 PD of fusional convergence and 10 PD of fusional divergence (Rowe, 2010). The average fusional convergence break point values in this study were lower than normal values. Similar to our results, lower mean values for fusional convergence were found by others (Jiménez et al., 2004, Etezad Razavi, 2010). It is accepted that in healthy subjects the fusional amplitudes vary considerably (Von Noorden, 1996).

In the present study, convergence insufficiency may have caused a deviation towards exophoria. Lavrich (2010) reported that most subjects with convergence insufficiency demonstrated varying degrees of exophoria or even an intermittent exotropia at near, and some subjects had reduced stereo acuity at near. With distance fixation a small exophoria or orthophoria was observed. Our results support previous research data that indicate a positive effect of using the vergence/accommodative exercise for improving convergence function (Rawstron et al., 2005; Aziz et al., 2006). Horwood et al. (2014) confirmed beneficial effects in testing of convergence function in an asymptomatic typical young adult population after a 2 week period of an intensive training program of various eye exercises (accommodation, vergence, convergence in excess of accommodation, accommodation in excess of convergence). Under controlled experimental conditions using exercises stressing more convergence than accommodation function, the most significant effect was observed after vergence exercises to a nonaccommodative target. Zupan et al. (2006) used quoits sheets in sports vision training in intercollegiate athletes to test the limits of both the accommodative and vergence systems. Training sessions ranged from 0 to over 80 for athletes. After more than 60 training sessions, the average break point value in convergence function increased by 73%. After maximal training sessions the results of divergence functions showed a 34% improvement in the break point. Similar to our study, Zupan et al.’s (2006) results indicated more susceptibility to training convergence than divergence function.

In contrast, Wood and Abernethy (2001) reported that a 4 week vision training program (enhancing visual and motor performance for racquet sports) was ineffective in improving vergence as well as phoria function. The authors suggested that training using a sports vision program and eyerobics video-based training had no long-term impact on ocular muscle balance. However, our study’s procedures when compared to the ones of Wood and Abernethy (2001) had different designs regarding the total duration of the intervention program. Similar to our procedures, other studies using periods of six, eight and more weeks of training showed beneficial effects on trained visual functions (Quevedo et al., 1999; Zupan et al., 2006; Maman et al., 2011; Rezaee et al., 2012; Schwab and Memmert, 2012). In addition, it is worth emphasizing that not all forms of ‘vision therapy’ are recommended to improve oculomotor functions. The most current regime to improve the balance of the co-acting pairs of extraocular muscles is to use an orthoptic program with a series of exercises including saccades, pursuits, base-in and base-out stereograms, jump ductions and accommodation (Lavrich, 2010).

It seems that one of the most important factors supporting the effectiveness of our program consisted in conducting the vision exercises under optimal conditions. The experimental group was relatively small and at all times controlled by a vision improvement teacher. The benefit of such vision training was likely due to the subject getting more encouragement and reinforcement to try harder (Horwood et al., 2014). It has been shown that receiving controlled therapy had a better effect compared to, for example, home-based methods (Scheiman, 2005). Additionally, Fray (2013) indicated that lower levels of alertness affected vergence fusion ranges. On the other hand, a relatively small number of participants in our experiment may be a limitation of this study. Future research should attempt to increase the number of participants, increase the frequency of testing the intervention and control group, and take into account placebo effects in order to obtain repetitive and more objective results. Additionally, it is important to explore how enhancement in visual function can influence specific motor performance in subjects practicing different sports disciplines.

The present study does offer some insight into the mechanisms explaining adaptive changes in visual function. In relation to the visual demands of different sports, the strength and flexibility of the vergence function is very important for the stability of athlete’s perception, especially during physical fatigue.

In summary, exophoria was the most frequent disorder of binocular vision revealed during the screening tests. Simultaneously, a low fusional vergence range was observed. The positive results obtained after a period of eight weeks of training suggest that binocular function, e.g. ocular alignment and motor fusion range, is trainable and can be improved by means of appropriate visual training. Further studies are needed to assess the current outcomes.

## Figures and Tables

**Table 1:** The pre-test results of distance and near dissociated horizontal phoria (n=24)

dissociated horizontal phoria	eye	ortophoria	Shift towards	

			esophoria	exoforia
		
		N (N%) Mean ±SD (PD)		
distance	RE	4 (16.67)	7 (29.16)	13 (54.17)
		0 ±0	1.33 ±1.09	0.93 ±0.60
	LE	7 (29.16)	6 (25)	11 (45.84)
		0 ±0	1.32 ±0.68	0.91 ±0.55
near	RE	3 (12.5)	1 (4.17)	20 (83.33)
		0 ±0	-	2.34 ±0.57
	LE	4 (16.67)	1(4.17)	19 (79.17)
		0 ±0	-	2.17 ±1.09

RE – right eye, LE - left eye

**Table 2:** The pre-test results of motor foria (n=24)

Motor fusion	Mean ±SD (degrees of arc)	Approximately Mean ±SD (PD)	N (%) below normal range (25 PD-10 PD)
convergence	12.38 ±8.20	24.76 ±16.08	58.34
divergence	4.74 ±1.93	9.48 ±3.79	41.67
